# Simulation shows undesirable results for competing risks analysis with time-dependent covariates for clinical outcomes

**DOI:** 10.1186/s12874-018-0535-5

**Published:** 2018-07-16

**Authors:** Inga Poguntke, Martin Schumacher, Jan Beyersmann, Martin Wolkewitz on behalf of COMBACTE-MAGNET Consortium

**Affiliations:** 1 Institute for Medical Biometry and Statistics, Faculty of Medicine and Medical Center – University of Freiburg, Stefan-Meier-Str. 26, Freiburg, 79104 Germany; 2 Institute of Statistics, Ulm University, Helmholtzstr. 20, Ulm, 89081 Germany

**Keywords:** Time-dependent covariates, Subdistribution approach, (Internal) left-truncation, Fine and gray model

## Abstract

**Background:**

We evaluate three methods for competing risks analysis with time-dependent covariates in comparison with the corresponding methods with time-independent covariates.

**Methods:**

We used cause-specific hazard analysis and two summary approaches for in-hospital death: logistic regression and regression of the subdistribution hazard. We analysed real hospital data (n=1864) and considered pneumonia on admission / hospital-acquired pneumonia as time-independent / time-dependent covariates for the competing events ’discharge alive’ and ’in-hospital death’. Several simulation studies with time-constant hazards were conducted.

**Results:**

All approaches capture the effect of time-independent covariates, whereas the approaches perform differently with time-dependent covariates. The subdistribution approach for time-dependent covariates detected effects in a simulated no-effects setting and provided counter-intuitive effects in other settings.

**Conclusions:**

The extension of the Fine and Gray model to time-dependent covariates is in general not a helpful synthesis of the cause-specific hazards. Cause-specific hazard analysis and, for uncensored data, the odds ratio are capable of handling competing risks data with time-dependent covariates but the use of the subdistribution approach should be neglected until the problems can be resolved. For general right-censored data, cause-specific hazard analysis is the method of choice.

**Electronic supplementary material:**

The online version of this article (10.1186/s12874-018-0535-5) contains supplementary material, which is available to authorized users.

## Background

Competing events occur frequently in time-to-event studies in clinical epidemiology [1]. For instance, discharge is a competing event for hospital death since discharged patients are usually in a better health condition (with improved survival) than hospitalized patients. It is common that there are time-dependent covariates in combination with competing events. A widely discussed topic is hospital-acquired infections (a time-dependent covariate) and their effect on the risk of dying in the hospital (discharge as a competing event) (see e.g. [2]). The first step in such settings is to conduct a cause-specific hazard (CSH) analysis and to infer the effect from these findings.

The subdistribution by Fine and Gray is commonly used to determine how one of many competing outcome probabilities is affected by time-independent covariates [3]. The subdistribution offers an easy way to receive a summary analysis of all CSH’s and reestablishes the useful one-to-one relationship with the cumulative incidence function. For time-dependent covariates, the analysis gets more involved and the standard model by Fine and Gray does not hold anymore [4]. Beyersmann and Schumacher offered a solution for using the subdistribution in a time-dependent framework [5]. This approach and further extensions are used in current literature to assess the influence of a time-dependent covariate on the event of interest [6–9].

In this paper, we consider a hospital setting with pneumonia on admission/hospital-acquired pneumonia (HAP) as time-independent/time-dependent covariates for the competing events ’discharge alive’ and ’in-hospital death’ with time-constant hazards. First, we present our real data example and reassess the benefits of a CSH analysis and two types of synthesis of the cause-specifc analyses: the logistic regression and the regression of the subdistribution hazard by Fine and Gray in a time-independent framework. We profit here from the specific situation in hospital epidemiology where survival analysis is used to investigate time-dependent events, but where follow-up is essentially complete. Thus, logistic regression may be applied.

Then, we describe the CSH analysis, the logistic regression, and the subdistribution approach by Beyersmann and Schumacher applied in this time-dependent scenario thoroughly. We give a summary table of the expected properties in both a time-independent and a time-dependent framework. For further understanding, we conduct several simulation studies to study the performance more thoroughly. In conclusion, we summarize the findings of this paper as well as discuss the properties and results from the simulation study of the different approaches.

## Methods

**Fig. 1:**
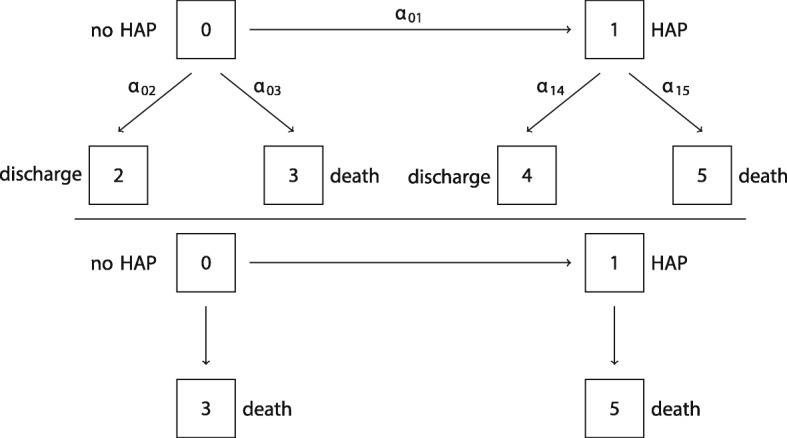
The multistate model including the binary time-dependent covariate hospital-acquired pneumonia and the corresponding model analyzed in the subdistribution approach (bottom). This holds for the time-independent pneumonia on admission analogously if one removes the transition from state 0 to state 1

As already mentioned, a common example of a time-dependent competing risks setting is a hospital-acquired infection, e.g. pneumonia, and its effect on hospital death and discharge alive. Therefore, we will consider a binary time-independent covariate with values in {0, 1}, incorporating pneumonia on admission in the following way: 0 as the absence of a pneumonia (risk factor) and 1 as the presence of pneumonia (risk factor). Note, that we will not model recovery. In other words, the covariate value will not change back to 0 when the patient recovers from pneumonia and patients in state 1 are in-hospital pneumonia patients in the sense that pneumonia has occured in the past. The competing events are death in the hospital (3, 5 respectively) and discharge alive (2, 4 respectively). Additionally, no censoring will be modeled. This mimics a realistic setting in hospital data, since censoring is in general uncommon. Most of the patients will be followed until their death in the hospital or until their discharge (alive). The censoring is exclusively administrative and typically negligible. The corresponding 6-state multistate model in a time-dependent setting can be seen in Fig. 1(top). The bottom part displays the corresponding subdistribution process.

### Real data example

The data set is from the SIR 3 (Spread of nosocomial Infections and Resistant pathogens) cohort study conducted at the Charité university hospital in Berlin, Germany [10]. Patients were followed until discharge or death in the hospital; there was no follow-up beyond discharge. It is the same data set that Beyersmann and Schumacher used in their paper to study the usefulness of the subdistribution approach [5].

For illustrative purposes, we removed the censored individuals (n =30 (1.6%)) to obtain a complete data set that suits our assumed setting. This is why our results differ slightly from the ones obtained by Beyersmann and Schumacher. The impact of pneumonia on admission (time-independent) and HAP (time-dependent covariate) on the risk of dying in the hospital is investigated; discharge alive is the competing event for in-hospital death as discharged patients have an improved survival compared to hospitalized patients.

In the data set, there were 1876 admissions to the hospital, 30 of those were censored, which leaves 1846 patients in the data set. Two hundred eight patients were admitted with pneumonia of which 160 were discharged alive. The remaining 48 patients died in the hospital. In total, 1638 patients were admitted without pneumonia. Of these, 1472 were discharged alive and 166 died in the hospital.

With respect to HAP, of the uncensored 1846 individuals, 151 developed a pneumonia in the hospital. Of those, 33 died in the hospital and 118 were discharged alive. Of the 1695 patients who didn’t acquire pneumonia in the hospital, 1514 patients were discharged alive and 181 patients died during their stay.

### Simulated data

For studying the performance of the different approaches, we used the following simulation technique. Throughout this paper, we assume constant hazards. In the simulation studies we simulated 100 data sets with 10000 individuals to see if the methods provide accurate results on average. For the simulation of time-independent covariates, we have a classical competing risks setting with two risk factor groups (’pneumonia on admission’ yes or no). For illustration, we assume time-constant hazard rates. Thus we have following hazards: discharge hazard of patients without pneumonia on admission (*α*_02_), in-hospital death hazard of patients without pneumonia on admission (*α*_03_) and the corresponding hazards (*α*_14_, *α*_15_) for patients with pneumonia on admission. We simulated data according to the procedure in the book by Beyersmann [11]. That is, for patients without pneumonia, length of stay was simulated with an all-cause hazard *α*_02_+*α*_03_, resulting in in-hospital death with probability $\frac {\alpha _{03}}{(\alpha _{02} + \alpha _{03})}$.

For the time-dependent setting, we simulated individuals in the 6-state multistate model as a nested sequence of competing risks processes as described in [11]. That is, all patients start in state 0 and we simulate the first transition as a competing risk experiment between states 2, 3, and 1 with all-cause hazard *α*_01_+*α*_02_+*α*_03_ for simulation of length of stay, e.g. resulting in a pneumonia with probability $\frac {\alpha _{01}}{(\alpha _{01} + \alpha _{02} + \alpha _{03})}$. Since the states 2 and 3 are absorbing, the experiment is over for all patients that make a transition into one of these states. If however, the patient moved into state 1, we conduct another competing risks experiment between states 4 and 5 with an all-cause hazard *α*_14_+*α*_15_. Since both states are absorbing, the simulation ends, once all individuals make a transition into one of these states. The R code used for simulation and analysis of the data is supplied as Additional file 1.

### Competing risk analysis for time-independent covariates

#### Cause-specific hazard analysis for discharge (alive) and in-hospital death

The classical approach to investigate the binary time-independent covariate "pneumonia on admission" would be to conduct a CSH analysis for both competing events, death and alive discharge. Then, one would compare the hazard ratios (HR) of both events in order to assess the impact of the covariate. For time-constant hazards, it is

$$\text{HR(death)} =\frac{\alpha_{15}}{\alpha_{03}}\text{and}\ \text{HR(discharge)} =\frac{\alpha_{14}}{\alpha_{02}} $$

for pneumonia vs. no pneumonia on admission. Such a cause-specific analysis gives insights into effects on the instantaneous (i.e. daily) risk of dying in the hospital and being discharged from the hospital. Both HR’s have to be interpreted side-by-side for each risk factor. As it is an *instantaneous* risk approach and discharge is a competing event for in-hospital death, it provides no direct insights into the *cumulative* risk of dying in the hospital. Cumulative approaches are presented in the following two sections.

#### Logistic regression for in-hospital death

In a simple logistic regression model, one studies the effect of the binary time-independent covariate pneumonia on admission on the cumulative risk of in-hospital death *at the end of follow-up*. In other words, the odds ratio (OR) compares the cumulative risk of in-hospital death on the plateau of the cumulative incidence functions. The odds for in-hospital death at the end of follow-up for patients with pneumonia on admission is given through

$${{} \begin{aligned} \text{Odds(death}|\text{pneumonia on adm)} &= \frac{P(\text{death}|\text{pneumonia on adm})}{1-P(\text{death}|\text{pneumonia on adm})}\\ &= \frac{\alpha_{15}}{\alpha_{14}+\alpha_{15}}:\frac{\alpha_{14}}{\alpha_{14}+\alpha_{15}}\\ &= \frac{\alpha_{15}}{\alpha_{14}} \end{aligned}} $$

and the odds for in-hospital death at the end of follow-up for patients without pneumonia on admission is

$${{} \begin{aligned} \text{Odds(death}|\text{no pneumonia on adm)}&= \frac{P(\text{death}|\text{no pneumonia on adm})}{1-P(\text{death}|\text{no pneumonia on adm})}\\ &=\frac{\alpha_{03}}{\alpha_{02}+\alpha_{03}}:\frac{\alpha_{02}}{\alpha_{02}+\alpha_{03}} \\ &= \frac{\alpha_{03}}{\alpha_{02}} \end{aligned}} $$

as can be seen in [2, 12]. Then, the OR in our scenario with constant hazards is the quotient of the HR of the event of interest, death, and the one for the competing event, discharge, and resolves to

$$ {{} \begin{aligned} \text{OR(death)} &=\frac{P(\text{death}|\text{pneumonia})}{1-P(\text{death}|\text{pneumonia})} : \frac{P(\text{death}|\text{no pneumonia})}{1-P(\text{death}|\text{no pneumonia})}\\ &= \frac{\alpha_{15}}{\alpha_{14}} : \frac{\alpha_{03}}{\alpha_{02}}\\ &=\frac{\text{HR(death)}}{\text{HR(discharge)}}. \end{aligned}} $$

An OR greater than 1 implies a higher risk of experiencing the event of interest, here in-hospital death, for patients with the risk factor pneumonia. Contrarily, an OR smaller than 1 connotes a lower risk of in-hospital death for patients with pneumonia on admission. Looking at the formula of the OR, we see that both cause-specific HR’s are involved. For example, an HR for discharge of less than 1 and an HR for death of 1 would lead to an OR greater than 1 and to the interpretation of a higher risk of dying for patients with pneumonia on admission.

Summarizing, the OR together with the CSH analysis is able to capture both indirect (effects through the competing hazard) and direct effects of the covariate if one additionally considers the baseline hazard. It has a probability interpretation and an easy-to-communicate way to assess the effect. It is not a direct probability measure, but a function thereof. Yet, it does not allow for censoring, does not account for time-to-event, and is only able to display effects on a cumulative incidence function on a plateau.

#### The subdistribution approach for in-hospital death

The subdistribution by Fine and Gray offers an easy way to receive a summary analysis of all CSH’s by introducing a subdistribution hazard [3]. The subdistribution hazard is the hazard that is directly linked to the cumulative incidence function. For estimation, Fine and Gray stop individual trajectories with the occurence of a competing event. In other words, the patients experiencing a competing event stay in the risk set forever. This leads to the subdistribution hazard being smaller than the CSH since the risk set is artificially inflated. The higher the competing hazard, the more patients experience a competing event and the more the subdistribution hazard is lowered in comparison to the CSH.

To analyze the effect of a covariate, one would look at the subdistribution hazard ratio (SHR) of the event of interest, here death, and get a direct reflection of the effect of the covariate on the probability of the event of interest. A SHR of greater than 1 would imply a higher risk of dying in the hospital with pneumonia on admission. A SHR of 1 would imply no effect and a SHR of less than 1 connotes a lower risk of dying in the hospital with pneumonia on admission. It is an easy-to-communicate summary analysis of all CSH’s. The SHR quantifies the effect which is seen in the cumulative incidence functions (over the time in hospital). A crucial point of criticism is the lack of a exact interpretation and the misspecificcation cause through proportional CSHs not implying proportional subdistributions in general [13, 14]. The properties of the subdistribution in comparison with logistic regression are summarized in Table 1. Next, we will explain the analysis when time-dependent covariates are involved.

**Table 1:** Overview of the properties of logistic regression (LR) and the subdistribution models in a time-independent and time-dependent setting

Property	Time-independent		Time-dependent	
	Fine&gray	LR	Beyersmann & Schumacher	LR
Allowance for censoring	Yes	No	Yes	No
Accounting for time-to-event	Yes	No	Yes	No
Ability to display cumulative incidence functions	Yes	Only plateau	No	Only plateau
Interpretation	Challenging	See text	Challenging	See text
Probability interpretation	Yes	Yes	No	Yes
Dependency on the infection hazard	.	.	Yes	No
Simulation performance:				
Ability to capture no effect on cause-specific hazards (*α*_15_=*α*_03_,*α*_14_=*α*_02_)	Yes	Yes	No	Yes
Ability to capture negative effect on death hazard (*α*_15_>*α*_03_,*α*_14_=*α*_02_)	Yes	Yes	Yes, magnitude difficult to interpret	Yes
Ability to capture positive effect on death hazard (*α*_15_<*α*_03_,*α*_14_=*α*_02_)	Yes	Yes	Questionable	Yes
Ability to capture negative effect on discharge hazard (*α*_15_=*α*_03_,*α*_14_<*α*_02_)	Yes	Yes	Questionable	Yes
Ability to capture positive effect on discharge hazard (*α*_15_=*α*_03_,*α*_14_>*α*_02_)	Yes	Yes	Questionable	Yes

### Competing risk analysis for time-dependent covariates

We will now consider HAP as a time-dependent binary covariate. This again includes a multistate model with two competing risks, death and discharge (alive), and only one binary time-dependent covariate, HAP (see Fig. 1 top). A patient is admitted to the hospital and can either be discharged alive or die (with or without experiencing an HAP). Thus, we additionally obtain the hazard *α*_01_ to acquire HAP.

#### Cause-specific hazard analysis for discharge (alive) and in-hospital death

As for time-independent covariates, the CSH analysis should always be part of the investigation [15]. This includes looking at the HR’s for death and discharge. Analogously, separate Cox models will be applied to each competing event with the only difference that the covariate is now time-dependent. See the time-independent case for details on the interpretation of the results of the Cox model.

#### Logistic regression for in-hospital death

Again, *at the end of hospital stay* means that the internal time-dependency within the hospital is ignored. We denote *P*(in-hospital death|with HAP) and *P*(in-hospital death|without HAP) as the probability of in-hospital death *at the end of hospital stay* for patients with and without an HAP, respectively. As HAP develops over time, these probabilities are not simple. According to Cube et al. [12], with *α*_0_=*α*_01_+*α*_02_+*α*_03_ and *α*_1_=*α*_14_+*α*_15_ and the state occupation probabilities *P*_0*j*_(*t*)=*P*(in state j at time t), the respective time-dependent versions are

$${ \begin{aligned} &P(\text{in hospital-death in} [0,t] | \text{with HAP in} [0,t])\\ &= \frac{P_{05}(t)}{P_{01}(t) + P_{04}(t) + P_{05}(t)}\\ &= \frac{\alpha_{15}}{\alpha_{1}(\alpha_{1}-\alpha_{0})} \frac{\alpha_{1}-\alpha_{0} - \alpha_{1}\cdot \exp (-\alpha_{0}t) + \alpha_{0}\cdot \exp (-\alpha_{1}t)}{1 - \exp(-\alpha_{0}t)} \\ \end{aligned}} $$

and

$$\begin{array}{*{20}l} &P(\text{in hospital-death in} [0,t] | \text{without HAP in} [0,t])\\ &=\frac{P_{03}(t)}{P_{00}(t) + P_{02}(t) + P_{03}(t)}\\ &= \frac{\alpha_{03}(1 - \exp (-\alpha_{0}t))}{\alpha_{01}\exp (-\alpha_{0}t) + \alpha_{02} + \alpha_{03}}.\\ \end{array} $$

For time-constant hazards, Cube et al. recently showed [12] that these become the limits of their *time-dependent* versions and resolve to

$$\begin{array}{*{20}l} P(\text{in-hospital death}|\text{with HAP})&=\alpha_{15}/(\alpha_{14}+\alpha_{15})\\ \end{array} $$

and

$$\begin{array}{*{20}l} P(\text{in-hospital death}|\text{without HAP})&=\alpha_{03}/(\alpha_{02}+\alpha_{03}).\\ \end{array} $$

With similar arguments as in the time-independent setting, this includes analyzing the OR for the event of interest, i.e. death,

$${{}\begin{aligned} \text{OR (in-hospital death)} &=\frac{\alpha_{15}}{\alpha_{03}} \times \frac{\alpha_{02}}{\alpha_{14}} = \frac{\text{HR(in-hospital death)}}{\text{HR(discharge)}}, \end{aligned}} $$

which consists of analyzing the quotient of the two death odds

$$\begin{array}{*{20}l} \text{Odds of in-hospital death (with HAP)}&=\alpha_{15}/\alpha_{14} \end{array} $$

and

$$\begin{array}{*{20}l} \text{Odds of in-hospital death (without HAP)}&=\alpha_{03}/\alpha_{02} \end{array} $$

[2, 12]. Logistic regression does not allow for censoring. This is not an acute issue in hospital data since there usually is very little censoring, but has to be taken into account in more general settings. The OR is easy to analyze and the interpretation of the OR stays the same as in the previous sections. The OR is a summary analysis of all CSHs and catches the effect of the time-dependent covariate very well. The properties can also be seen in Table 1. Other than the fact that logistic regression does not allow for censoring, it has many of the perks desired in an approach in a time-dependent competing risks framework. Summarizing, it offers an easy-to-communicate summary analysis which is independent of the infection hazard and has a probability interpretation. It is questionable whether not including the infection hazard holds enough information to describe a time-dependent setting with a plausible probability interpretation. As we will see later, including the infection hazard might also be problematic.

#### The subdistribution approach for in-hospital death

In 2008 [5] Beyersmann and Schumacher extended the Fine and Gray model to time-dependent covariates by modeling competing events and the values of a categorical time-dependent covariate in one multistate model. Even though the cumulative incidence function for time-dependent covariates is not clearly defined, the aim of this approach is to achieve a synthesis of the CSH analysis. The idea of Beyersmann and Schumacher is to transform the 6-state multistate model in Fig. 1 (top) into a subdistribution-type model that can be seen in Fig. 1 (bottom) corresponding to the scenario in the time-dependent setting. The subdistribution-type process (bottom) leaves individuals who experience a competing event in the risk set of the previous occupied state. In our example, an individual would stay in state 0 or 1 after experiencing a competing event. This alters the risk sets considerably, keeping individuals with a competing event at risk forever as in the Fine and Gray approach, and will be a topic of a discussion later on. This means that the subdistribution will be composed of state 0 and state 1 with the difference that a transition out of state 0 and into state 1 is possible, that is late entry in state 1 and exit to a non-absorbing state from state 0. An issue resulting from this is the risk set in state 1 has to build up first. The covariate process will be stopped at the last known value. As a consequence of this, the same covariate is analyzed in a standard Cox model and in an extended Fine and Gray model, that is the stopped covariate process *Z*↦*Z*(*t*∧*T*), where *t*∧*T* is the minimum of the time *t* and the length of stay *T*. Therefore, Beyersmann and Schumacher introduced a time-dependent covariate in a competing risks setting by combining the multistate with the subdistribution framework. This approach provides one quantity, the SHR, that measures the impact of the covariate on the event of interest directly and is easy to communicate to statisticians and non-statisticians. Analogous to the time-independent setting, the exact interpretation remains unresolved and is the subject of ongoing discussion [13]. Furthermore, proportional CSH’s do not in general imply proportional subdistributions [14].

## Results

First, we analyzed the SIR 3 data set according to the three different approaches with respect to pneumonia on admission and additionally with respect to HAP. The results are summarized in the upper part of Table 2. The estimated constant hazards can also be found in this table. The results of the simulation study will be presented on the bottom of this table and the resulting properties will be compared in Table 1.

**Table 2:** Results in the SIR 3 and simulated data sets

Type		Pneumonia on admission	Hospital-acquired pneumonia
Estimated constant hazards in the data sets:			
Infection hazard *α*_01_	-	0.0063	
Discharge hazard w/o pneumonia *α*_02_	0.0671	0.0627	
Death hazard w/o pneumonia *α*_03_	0.0076	0.0075	
Discharge hazard with pneumonia *α*_14_	0.0279	0.0334	
Death hazard with pneumonia *α*_15_	0.0084	0.0093	
Results:			
HR(death)	1.11;1.24^∗^	1.00 (0.72,1.39)	0.9 (0.6,1.34)
HR(discharge)	0.42;0.53^∗^	0.44 (0.38,0.52)	0.59 (0.49,0.72)
SHR(death)		2.37 (1.72,3.26)	3.44 (2.36,5.03)
OR(death)	2.64;2.34^∗^	2.66 (1.86,3.81)	2.34 (1.54,3.54)
Simulated data:			
Scenario 0: *α*_01_=0.05, *α*_02_=0.2,*α*_14_=0.1, *α*_03_=*α*_15_=0.05			
HR(death)	1	1.00 (0.92,1.12)	0.99 (0.91,1.09)
HR(discharge)	0.5	0.50 (0.48,0.53)	0.50 (0.47,0.53)
SHR(death)		1.73 (1.59,1.93)	3.24 (2.99,3.55)
OR(death)	2	2.01 (1.81,2.27)	1.99 (1.77,2.26)
Scenario 1: *α*_01_=0.01, *α*_02_=*α*_14_=0.05, *α*_03_=*α*_15_=0.01			
HR(death)	1	1.01 (0.90,1.15)	1.00 (0.87,1.15)
HR(discharge)	1	1.00 (0.95,1.05)	1.00 (0.94,1.06)
SHR(death)		1.01 (0.9,1.17)	1.98 (1.77,2.26)
OR(death)	1	1.01 (0.89,1.18)	1.00 (0.89,1.15)
Scenario 2: *α*_01_=0.05, *α*_02_=*α*_14_=0.2, *α*_03_=0.05, *α*_15_=0.1			
HR(death)	2	2.01 (1.86,2.22)	1.98 (1.82,2.18)
HR(discharge)	1	1.00 (0.95,1.06)	1.00 (0.95,1.05)
SHR(death)		1.84 (1.68,2.05)	3.57 (3.26,3.92)
OR(death)	2	2.01 (1.81,2.27)	1.99 (1.77,2.26)
Scenario 3: *α*_01_=0.05, *α*_02_=*α*_14_=0.2, *α*_03_=0.1, *α*_15_=0.075			
HR(death)	0.75	0.75 (0.7,0.82)	0.75 (0.67,0.86)
HR(discharge)	1	1.00 (0.95,1.05)	1.00 (0.93,1.08)
SHR(death)		0.78 (0.72,0.86)	1.50 (1.36,1.70)
OR(death)	0.75	0.75 (0.68,0.84)	0.75 (0.66,0.86)
Scenario 4: *α*_01_=0.05, *α*_02_=0.2, *α*_14_=0.3, *α*_03_=*α*_15_=0.05			
HR(death)	1	1.01 (0.91,1.16)	0.99 (0.88,1.11)
HR(discharge)	1.5	1.50 (1.42,1.57)	1.50 (1.43,1.59)
SHR(death)		0.71 (0.63,0.83)	1.40 (1.27,1.58)
OR(death)	0.67	0.68 (0.60,0.80)	0.66 (0.60,0.75)

Upper part: Estimated hazards and results in the SIR 3 data set 95% confidence intervals are given in parenthesis. Lower part: Average results of 100 data sets with 10000 individuals per data set with empirical 95% confidence intervals in parenthesis. If applicable, the true HRs and ORs are given. The infection hazard *α*_01_ is only applicable for HAP. ^∗^: The values show the HRs computed with the estimated constant hazards. The first value corresponds to pneumonia on admission, the second to HAP

### Pneumonia on admission

The Cox analysis of the CSH’s showed no effect of pneumonia on admission on the hazard to die in the hospital (HR(death) =1). Additionally, analysis showed a HR for discharge of 0.44. Patients with pneumonia on admission have a lower instantaneous (i.e., daily) chance of getting discharged alive compared with patients without pneumonia on admission and are therefore being exposed to the risk of dying in hospital for a longer time, each day anew. This indirect effect make patients with pneumonia on admission eventually die more frequently in the hospital compared with patients without pneumonia on admission. Thus, the cumulative risk to die in the hospital is indirectly increased due to their prolonged length of stay. It would be useful to receive a summary analysis via logistic regression or the SHR. The OR in this data set takes value 2.66, the SHR value 2.37, and they are both able to catch and summarize the effect of pneumonia on admission on the risk of death very well. Since the effect on the discharge hazard is pretty distinct, it is not surprising to have values higher than 2.

### Hospital-acquired Pneumonia

**Fig. 2:**
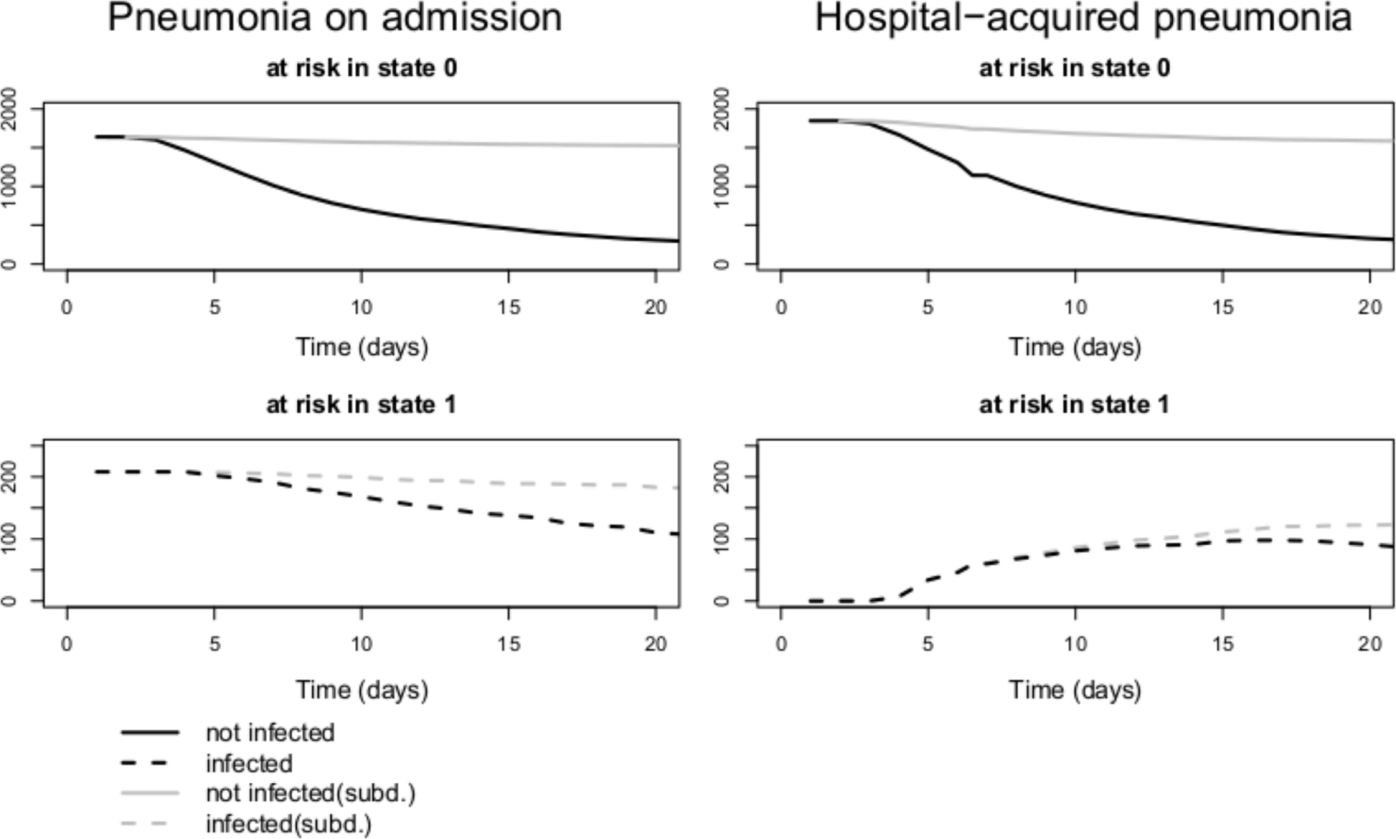
Risk sets in states 0 (solid lines) and state 1 (dotted lines) in the original multistate process (black lines) and the subdistribution process (grey lines) in the data of the SIR 3

The HR for death is 0.9 (the 95%- confidence interval includes 1) and the one for discharge takes value 0.59. Therefore, the situation is similar (even though less pronounced) to the one in the time-independent setting, but the risk sets behave differently, which we illustrated in Fig. 2. There is no significant effect on the death hazard, but the discharge hazard is reduced for individuals who experienced pneumonia in the hospital. The OR is comparable to the time-independent setting and takes value 2.34, whereas the SHR is considerably higher compared to the one in the time-dependent setting with a value of 3.44. This is surprising since the initial situation is similar in the two scenarios with respect to the hazards into the absorbing states. Furthermore, the SHR in the time-dependent setting compared to the one in the time-independent setting is considerably higher. We further investigated this unexpected result by simulation.

In Fig. 2, a clear difference in the risk sets can be seen immediately. In the time-independent setting, all patients already have their final covariate value, 0 or 1, and the risk sets only shrink during the process when patients make a transition into an absorbing state.

In the time-dependent setting, all individuals start in the intitial state 0, whereas the risk set in state 1 is empty and individuals have to make the transition into state 1 before they appear in the risk set and are then under the influence of the hazards of transitions out of state 1. Therefore, the curves of the risk sets in both the original multistate model and the subdistribution model are quite different, and the risk sets in state 0 and in state 1 change in a different manner by moving to the subdistribution process. The risk set in state 0 in the subdistribution approach is bigger than the corresponding risk set in the multistate model. In contrast, the risk sets of the infected individuals are almost equal just after the initial time.

### Simulation studies

First, we wanted to study a similar situation as the situation in the SIR 3 data in the time-independent and the time-dependent setting. This is why we simulated a new data set with those constant hazards that we approximately estimated from the SIR 3 study (Scenario 0). As we can see, the trend of the results does not change. The approaches capture the underlying true value in the time-independent setting very well and both the OR and the SHR produce similar results, which was already found by Beyersmann [16]. Again, the SHR is rather large.

Next, we wanted to mimic this setting in a no-effect model (Scenario 1). We would expect all of the effect measures to be 1 or at least the confidence intervals to include 1 independent of the time-dependency. As we can see, the time-independent setting delivers the expected results. In the time-dependent setting, the SHR takes value 1.91 and would lead to the false conclusion of HAP having a pronounced effect on the death risk, although we assumed a no-effect model.

This is an undesirable artefact and has to be investigated further. Therefore, we chose three additional scenarios (Scenario 2−4 in Table 2) with different tendencies of the effects. Scenario 2 only has an effect on the death hazard, which is higher for patients with either type of pneumonia. Again, the time-independent simulation showed the desired results as it will do in the next scenarios, whereas the SHR with a value of 3.57 is rather large. In scenario 3, pneumonia is set to have a slight effect on the death hazard, which is reduced out of state 1. Here, the SHR of the time-dependent setting does not produce a useful result, since it is returning a value that would lead to the false conclusion of pneumonia having a negative effect on the event of interest, death. Similar to scenario 3, scenario 4 has an indirect effect on the risk of dying, which is obtained via an increased discharge hazard for patients with pneumonia. Again, only the SHR in the time-dependent setting arises suspicion in leading to the false conclusion that pneumonia has a negative effect. These results are not plausible and leaves to the acute need for clarification of the problems.

We simulated data in a different initial model but obtained similar undesirable results. To further investigate the effect of a hazard on the misperformance, we altered its value around the initial value of the hazard while keeping the other hazards fixed as in the initial model and took a look at the returned SHR. Higher values of *α*_14_,*α*_02_ respectively, lead to less plausible, in the sense of closer to the real value 1, results, whereas higher values of *α*_15_,*α*_03_ respectively, lead to more plausible results. The results with varying infection hazard, *α*_01_, will be presented in the next section.

#### The infection hazard

With higher infection hazards, there is no clear trend in performance observable, though a very small infection hazard leads to more plausible results than with a distinct infection hazard. We investigated this with another simulation study, where only the infection hazard *α*_01_ was varied, whereas all others were kept constant at the values of the initial model. The idea was to downsize the left-truncation. Therefore, we cut the initial infection hazard of value 0.05 at a predefined time point. There is a tendency of values closer to value 1 for cutting the infection hazard sooner. The earlier the internal left-truncation is curtailed, the closer the setting is to the time-independent case. This indicated that the infection hazard is pivotal in the subdistribution approach with time-dependent pneumonia status. We summarized the properties in Table 1 and discuss the undesirable findings in the next section.

## Discussion

We found the subdistribution approach returning unexpected effects in a simple simulation study with constant hazards, whereas the simple ad-hoc logistic regression returned reasonable results. The subdistribution approach detected effects in a simulated no-effects setting and provided counter-intuitive effects in other settings.

To our knowledge, this misperformance did not arouse suspicion in hospital epidemiology. It is very common that hospital-acquired infections are associated with an decreased discharge hazard (*α*_14_<*α*_02_) and not associated with the death hazard (*α*_15_≈*α*_03_). One would therefore expect an indirect effect of hospital-acquired infections on hospital mortality due to an extended length-of-stay and prolonged exposure to the death hazard. Thus, a SHR >>1 would be expected and it is not surprising that the subdistribution approach was not questioned in that scenario. In that matter, a current article by van Vught et. al. arouse our attention [7], where the SHR was used for the investigation of the time-dependent covariate hospital-acquired infections and multiple medical interventions and seemed to mirror the underlying true values very well. This can be explained by a similar setting to our simulation study, where we reduced the infection hazard. Patients that are in the hospital for at least two days are included in the study. Since the patients are already in the hospital for two days before they are included in the study and most of the interventions, e.g. mechanical ventilation, happen before that time, the internal left-truncation is very limited. Most people that get an intervention during their stay are already in state 1 when they are recorded in the study and only few of them make the transition after they are included in the study. Since this setting is closer to a time-independent setting and the internal left-truncation is cut early, it is plausible that the SHR seems to capture the underlying true values very well as seen in our simulation study.

During simulations, the internal left-truncation aroused suspicion and this example would underline the effect of better results with an earlier curtailed left-truncation. As we saw, cutting the infection hazard earlier lead to more plausible results and explains why van Vught et al. found plausible subdistribution results.

Additionally internal left-truncation leads to a risk set shift, in which the risk sets in the initial states representing covariate values behave in a different manner with time-dependent covariates than they do with time-independent covariates especially in the beginning of the study (recall Fig. 2).

If the competing discharge hazard is very small, the subdistribution hazard is close to the CSH of death and therefore the subdistribution approach can capture the no-effect model more easily. Vice versa, the higher the death hazard, the more patients leave the risk set ultimately and the subdistribution and the CSH are closer. The results of the simulation study confirm these considerations as the SHR was more plausible with higher death hazards and lower discharge hazards, respectively.

These are strong indicators that the internal left-truncation and the resulting risk set shift might cause the misperformance of the subdistribution approach. A weighting technique by Geskus [17] delivered promising results when applied to the subdistribution approach. Right now, we are still working on the theoretical background to support our findings. In a no-effect model, the weighting produced plausible results and even for a model with a distinct effect of the time-dependent covariate the results were more plausible. It is a promising start but has to undergo more theoretical considerations before this weighted subdistribution approach can be used in clinical practice. Another adjusted SHR was used by Ong et. al. to approach this problem more implicitly [18].

Another issue that cannot be neglected is the misspecification of the model assumptions as already discussed by Grambauer et. al. [14]. The proportional hazards assumption for the CSH’s or the subdistribution hazards usually preclude each other. Grambauer et. al. showed that the analysis still offers an useful summary analysis even if the model is misspecified. The possible problems in the subdistribution approach caused by misspecification should not be neglected anyhow. However, the time-dependent case leads to more problems than already present in the time-independent case. We have not investigated the situation of multiple time-dependent covariates, yet conjecture that the difficulties translate to multiple regression.

Summarizing, the subdistribution approach has shown undesireable results in several simulation studies and the use of the approach should be neglected until this issue has been resolved. Alternatives and thorough discussion of time-dependent covariates in a competing risks setting can also be found in a paper by Cortese et. al. [19]. Since the theoretical background is not fully resolved yet, we strongly recommend avoiding the use of the subdistribution approach for assessing the effect of a time-dependent covariate. In hospital epidemiology, we recommend the use of logistic regression in combination with a CSH analysis instead. This recommendation expands to the case of non-constant hazards. It has useful properties (Table 1) and an easy-to-communicate interpretation. It holds the advantages that we would wish for in a model and the simulation performance is good. Since censoring is not a big issue in the setting of hospital-acquired infections, this lack of allowance for censoring can be accepted as long as no better alternatives are at hand.

## Conclusions

This manuscript shows that the proposed extension of the subdistribution approach to time-dependent covariates is in general not a helpful synthesis of the CSH analysis. The cause-specific hazards should be the primarily analyzed, potentially followed by logistic regression as a simple ad-hoc analysis in the presence of no censoring to synthesize the cause-specific hazard analyses. The use of the subdistribution approach for time-dependent covariates requires methodological improvements before application.

## Additional file

Additional file 1 Zip file of R code used in the analyses. (ZIP 8 kb)

## Abbreviations

CSH: cause-specific hazard
HAP: hospital-acquired pneumonia
HR: hazard ratio
OR: odds ratio
SHR: subdistribution hazard ratio
